# Impacts of contracted endodontic cavities compared to traditional endodontic cavities in premolars

**DOI:** 10.1186/s12903-020-01237-w

**Published:** 2020-09-07

**Authors:** Juan Xia, Weidong Wang, Zhengmao Li, Bingpeng Lin, Qian Zhang, Qianzhou Jiang, Xuechao Yang

**Affiliations:** 1 Department of Endodontology, Affiliated Stomatology Hospital of Guangzhou Medical University, Guangzhou key Laboratory of Basic and Applied Research of Oral Regenerative Medicine, Guangzhou, 510182 Guangdong China; 2 Department of Basic Science of Stomatology, Affiliated Stomatology Hospital of Guangzhou Medical University, Guangzhou key Laboratory of Basic and Applied Research of Oral Regenerative Medicine, Guangzhou, 510182 Guangdong China; 3 Department of Orthodontic, Affiliated Stomatology Hospital of Guangzhou Medical University, Guangzhou key Laboratory of Basic and Applied Research of Oral Regenerative Medicine, Guangzhou, 510182 Guangdong China; 4 Department of Oral Function and Prosthetic Dentistry, College of Dental Science, Radboud University Nijmegen Medical Centre, Philips van Leydenlaan 25, 6525EX Nijmegen, The Netherlands

**Keywords:** 3D-printed template, Contracted endodontic cavities, Instrumentation efficacy, Root canal filling, Fracture resistance

## Abstract

**Background:**

This study aims to compare the percentage of dentin removed, instrumentation efficacy, root canal filling and load at fracture between contracted endodontic cavities, and traditional endodontic cavities on root canal therapy in premolars.

**Methods:**

Forty extracted intact human first premolars were imaged with micro-CT and randomly assigned to the contracted endodontic cavity (CEC) or traditional endodontic cavity (TEC) groups. CEC was prepared with the aid of a 3D-printed template, canals were prepared with a 0.04 taper M-Two rotary instrument, and cavities were restored with resin. Specimens were loaded to fracture in an Instron Universal Testing Machine after a fatigue phase. The data were analyzed by the independent samples T test and Mann-Whitney U test, appropriate post hoc tests.

**Results:**

In the premolars tested in vitro, the percentage of dentin removed in the premolars with two dental roots in the CEC group (3.85% ± 0.42%) was significantly smaller (*P* < 0.05) than in the TEC group (4.94% ± 0.5%). The untouched canal wall (UCW) after instrumentation for TECs (16.43% ± 6.56%) was significantly lower (*P* < .05) than the UCW (24.42% ± 9.19%) for CECs in single-rooted premolars. No significant differences were observed in the increased canal volume and surface areas in premolars between the TEC and CEC groups (*P* > 0.05). CECs conserved coronal dentin in premolars with two dental roots but no impact on the instrument efficacy.

There were no differences between the CEC groups and the TEC groups in the percentage of filling material and voids (*P* > 0.05). In addition, the mean load at failure of premolars did not significantly differ between the CEC and TEC groups and there was no significant difference in the type of fracture (*P* > 0.05).

**Conclusion:**

The results of this study suggest that CEC could not improve the fracture resistance of the endodontically treated premolars. The instrumentation efficacy and the percentage of filling material did not significantly differ between CECs and TECs in premolars.

## Background

Endodontic treatment is a procedure that consists of several steps aiming to retain the normal function of the treated tooth or prevent or heal the periapical periodontitis. The principle of access and coronal cavity preparation is the straight-line pathways into root canals to enhance instrumentation efficacy and prevent complications [1, 2]. The treatment associated loss of tooth structure could undermine the biomechanical responses of the tooth [3], especially in endodontically treated teeth [4].

Today, materials and novel concepts, including the development of nickel-titanium instruments and the concept of minimally invasive endodontics (MIE), are rapidly changing. MIE is characterized by “systematic respect for the original tissue” and “preventing or treating disease with as little loss of original tissue as possible [5].

Contracted endodontic cavities (CECs), which were inspired by the concepts of MIE, emphasize endodontically treated tooth structure preservation, including pericervical dentin (PCD). The preservation of PCD is important for dental structure and is associated with a long-term survival benefit [6].

Three-dimensional (3D) printing is widely used for preoperative planning and procedure rehearsal [7], orthognathic surgery [8], custom prosthetic design [9], endodontics [10, 11] and surgical guidance [12]. Besides, it would be a useful educational tool for teaching and to enhance communication between the patient and doctors. 3D printing technology could achieve precise design, positioning, and good communication before the operation. Therefore, this technology has been applied in a clinic more extensively to achieve good treatment outcomes [13, 14], besides the accuracy and safety for 3D printed template have been proved [15, 16]. This research was aimed to design the 3D printed template for endodontic cavities and explore the clinical significance of 3D printed template in endodontics.

In this study, systematic measurement, including, the percentage of dentin removed, instrumentation efficacy, the increased canal volume and surface areas, the increased sectional area, the percentage of the filling material, and fracture resistance of premolars was conducted. We systematic compare the effect of the percentage of the filling material and fracture resistance of premolars with contracted endodontic cavities.

## Methods

### Selection of teeth

The present study was approved by the Ethics Committee of the Hospital of Stomatology, Guangzhou Medical University (KY-2017-012). Forty human first premolars from an orthodontic tooth extraction in the oral and maxillofacial surgery department were collected extracted, informed written consent was obtained from each patient. Soft and hard tissue residuals on the surfaces of the teeth were removed using an ultrasonic scaler. All teeth had a fully formed apex without any defects or cracks on the surface and had no history of restoration. A curvature of 0–20°, according to Schneider [17] on buccolingual and mesiodistal radiographs was selected. The selected teeth were of similar dimensions. The evaluation of the sample selection was done by computerized microcomputed tomography, All teeth were numbered and assigned into four groups (*n* = 10/ each group) according to the random number table. CEC groups, Group 1: single-rooted mandibular first premolars with one root canal, Vertucci’s classification typeI; Group 2: two-rooted maxillary first premolars with double root canals, Vertucci’s classification type IV. TEC groups, Group 3: single-rooted mandibular first premolars with one root canal, Vertucci’s classification typeI; Group 4: two-rooted maxillary first premolars with double root canals, Vertucci’s classification type IV. All the datas were evaluated using CBCT. There was no statistically significant difference (*P* > 0.05) in BL, MD, or tooth root length between the CEC and TEC groups. The teeth were kept in 1% chloramine T trihydrate at room temperature until use.

### Manufacture of 3D-printed template

**Fig. 1:**
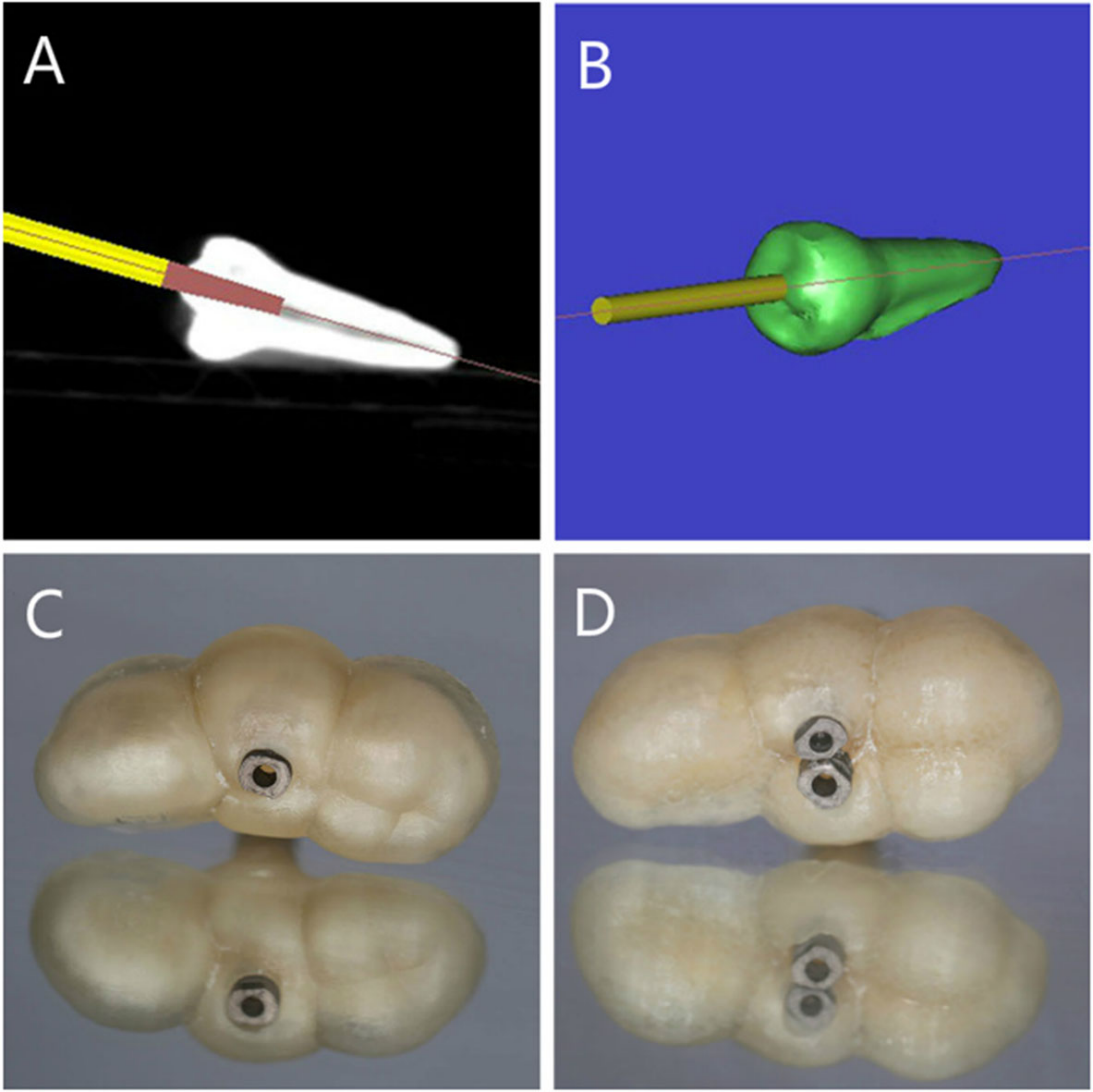
**a** The drill virtually superimposed on the tooth to create straight-line access to the apical third of the root canal. **b** A schematic of a drill in the tooth model. **c** A 3D-printed template positioned on the single-rooted premolar model. **d** Template positioned on the premolar with two dental roots

The guided access cavity was prepared using cone-beam computed tomography and optical surface scans. A high-resolution cone-beam computed tomography (CBCT) scan was taken to determine the exact location of the root canal. The drill was virtually superimposed on the root canal to plan the CEC outlines by projecting the access trajectory in each canal orifice that required the least tooth structure removal in Simplant (SIMPLANT, Materialise Dental, Leuven, Belgium) (Fig. 1). The data were then imported into Freeform (Geomagic Freeform, 3D Systems, Morrisville, North Carolina, USA). According to the location of the drill in Simplant, we made a guide template with straight-line pathways into the tooth canal. Additionally, we also designed a 3D printed cylindrical lampstand with a 0.2 mm gap to simulate the periodontal ligament for every specimen. The digitally designed template and 3D printed cylindrical lampstand were exported as STL-file and then were sent to a 3D printer (3D System 3510HB, 3D Systems, Morrisville, North Carolina, USA).

### Root canal preparation between TECs and CECs

First of all, the teeth were imaged with micro-CT (SkyScan 1172; Bruker micro-CT, Kontich, Belgium) imaging at 20 μm (pretreatment scan) to capture the original canal shape and volume of tooth tissue. In CEC preparation, a 3D-printed template was positioned on the tooth model (Fig. 1), and a guiding sleeve was placed on the hole.

CECs were drilled with long diamond burs (MANI SF-11, MANI INC, Japan) at high speed. The CEC access attested the distal and mesial accesses could be directed towards their respective orifices, which kept back the truss of dentin between the cavities. In the TEC group, conventional access cavities were prepared. After initial preparation with pathfile instruments (Dentsply Maillefer, Ballaigues, Switzerland), canals were prepared with 0.04 taper M-Two rotary instruments (VDW company, Munich, Germany) to size 35#. These instruments were used in a standard technique, The canals were irrigated with 3 ml of 5.25% sodium hypochlorite (Guangzhou Hui Fan company, Guangzhou, China) between use of each instrument, and then, each canal was irrigated with 5.25% sodium hypochlorite followed by irrigation for 30 s with ultrasonic oscillation tip (K15/21–25, SATELEC, France) coupled with an ultrasound device (SATELEC P5XS, Merignac, France) at power 7. After cleaning and shaping, the teeth were imaged again with micro-CT imaging at 20 μm (posttreatment scan) to capture the instrumented canal shape and volume of tooth tissue for comparative the differences. All canals were obturated with gutta-percha cones (Dentsply Sirona, New York, Pennsylvania, USA) and AH Plus sealer (Dentsply DeTrey, Konstanz, Germany). The thermoplastic continuous wave of condensation technique was used for obturation using a B&L-beta Gutta Percha Heating System (B&L Biotech, Inc., Korea). Smart Dentin Replacement (Dentsply, DE, USA) was used to imitate the lost dentin tissue, and 2 mm composite resin restorative material (SHOFU, Kyoto, Japan) was placed on the canal opening. The teeth were stored in physiological saline at 37 °C for 1 week. After that, each specimen was subjected to micro-CT imaging at 20 μm (finished scan).

### Load at fracture

**Fig. 2:**
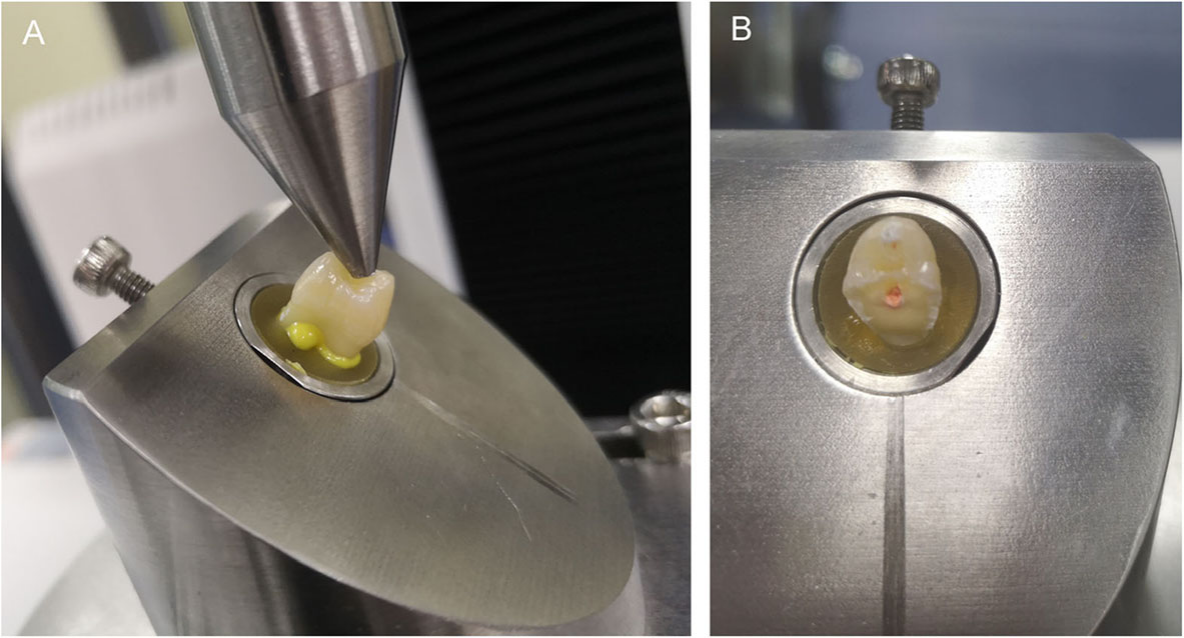
**a** Specimens placed in the Instron Universal Testing Machine. **b** The fractured tooth and the type of fracture was recorded

After root canal filling and micro-CT scanning, teeth with a 3D printed cylindrical lampstand were mounted in an Instron Testing machine (E3000, Instron, High Wycombe, UK). The specimens were subjected to 500000 loading cycles in the Instron Testing machine (E3000) axial forces, directed at a 135 angle from the long axis of the tooth [18], between 5 N–50 N at 15 HZ to simulate approximately 2 years of chewing function [19, 20]. After this fatigue phase, the specimens were placed in the Instron Universal Testing machine (E3366, Instron, MA, America). Each tooth was loaded at the central fossa at 135° from the tooth long axis to simulate a maximum bending motion of the tooth at buccal cervical areas [8]. A continuous compressive force was applied with a 2-mm spherical crosshead at 1 mm/min until failure occurred, which was defined as a 25% drop in the applied force [21] (Fig. 2a). The load at fracture was recorded in Newton (N), and the type of fracture was recorded (Fig. 2b).

### Evaluation methodology

**Fig. 3:**
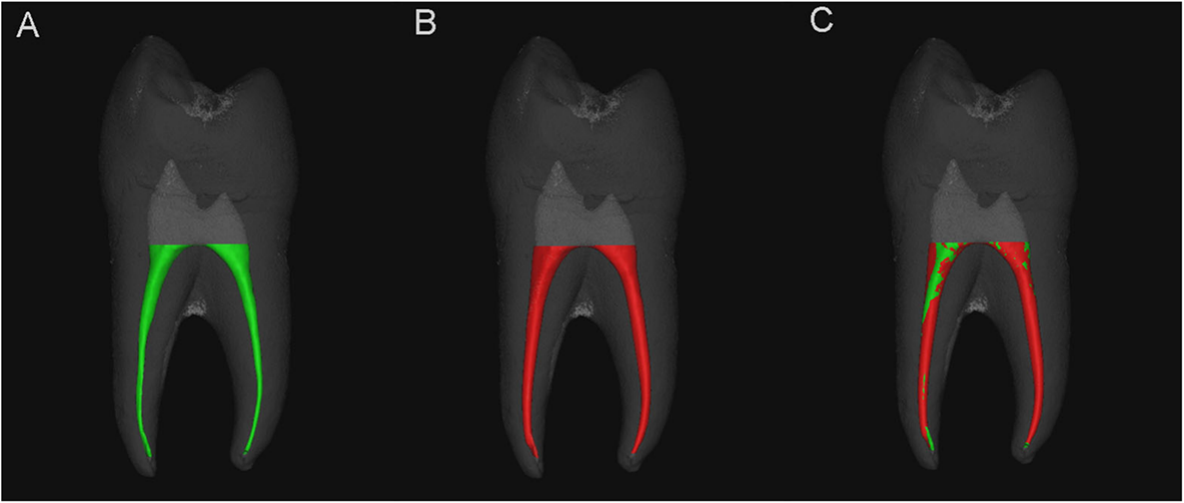
Preoperative (green) (**a**), postoperative (red), **b** and the aligned root canal (**c**) in 3-matic

**Fig. 4:**
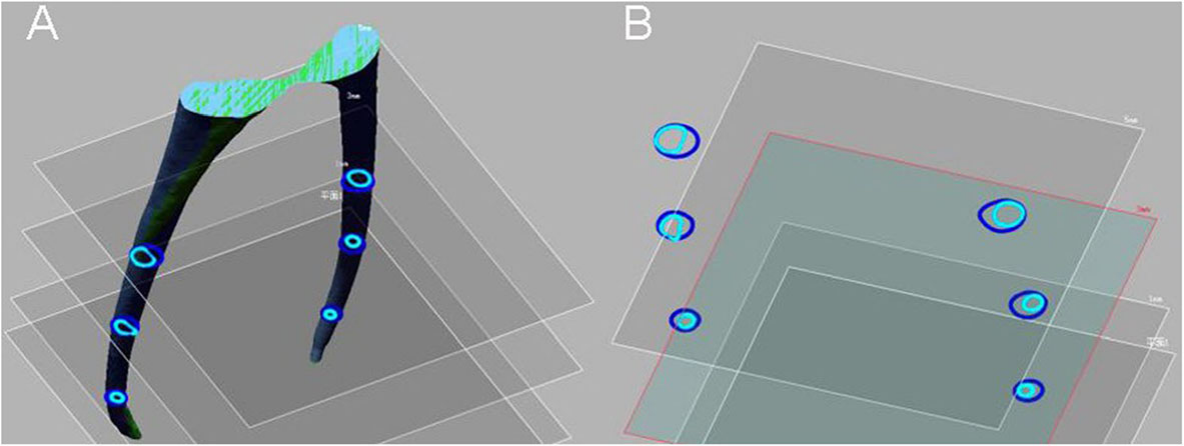
**a** The 1, 3, and 5 mm section from the apical. **b** The deviation of central point in Solid Work. Preoperative (aquamarine) and postoperative (mazarine)

After reconstruction with NRecon (Bruker micro CT, Kontich, Belgium) software, the volume of the tooth tissue was analyzed with CT-AN software (Bruker micro CT, Kontich, Belgium), we selected appropriate CT value as the segmentation of tooth volume. After the pretreatment scan and post-treatment scan were aligned in Data Viewer software (Bruker micro CT, Kontich, Belgium), the increased canal volume and surface areas after root canals shaped during the two different access opening procedures were measured using CT-An software. The proportion of untouched canal wall (UCW) in the canals was determined with 3-Matic (Fig. 3), and we measured the sectional section of 1, 3, and 5 mm from the apical and the deviation of the central point in Solid Work (Dassault, France) (Fig. 4). The percentage volume of root filling materials and any voids inside the region of interest were calculated in CT-An software, and all areas without filling within the root canal space were considered voids.

### Statistical analysis

The data were analyzed using IBM SPSS Statistics 16 software (Armonk, NY, USA), it was compared with independent samples T-tests, and the Mann-Whitney U test, *P* < .05 was considered significant.

## Results

The percentage of dentin removed in the premolars with two dental roots in the CEC group (3.85% ± 0.42%) was significantly smaller (*P* < 0.05) than in the TEC group (4.94% ± 0.5%). The UCW after instrumentation for TECs (16.43% ± 6.56%) was significantly lower (*P* < 0.05) than the UCW (24.42% ± 9.19%) for CECs in single-rooted premolars (Table 1). No significant differences were observed in the increased canal volume and surface areas in premolars between the TEC and CEC groups (*P* > 0.05). In the premolars with two dental roots, the increased sectional area of 1, 3, and 5 mm from the major apical foramen was significantly greater (*P* < 0.05) in the CEC group than in the TEC group. The deviation of the central point after instrumentation for TECs was significantly smaller (*P* < 0.05) than that for CECs. There was no significant difference (*P* > 0.05) between the TEC and CEC groups in the increased sectional area and the deviation of the central point in single-rooted premolars. Micro-CT analysis revealed that there were no differences between the CEC groups and the TEC groups in the percentage of filling material and voids (*P* > 0.05) (Table 2). In general, the mean load at failure of premolars did not significantly differ between the CEC and TEC groups, and there was no significant difference in the type of fracture (*P* > 0.05) (Table 3).

**Table 1:** Unmodified canal wall after CEC or TEC preparation and root canal instrumentation in premolars assessed by Micro-CT imaging

Tooth type (*n* = 10)	UCW (% of total canal wall surface)		***P*** value
	CEC	TEC	
Single-rooted premolars	24.42 ± 9.19%	16.43 ± 6.56%	0.038
Two dental root premolars	18.62 ± 5.85%	21.28 ± 8.91%	0.441

**Table 2:** Mean values ± standard deviations of the filling material and voids volume in premolars assessed by Micro-CT imaging

Groups	Region	Filling material (%)	Voids (%)
TEC (single-rooted premolars)	All	95.35 ± 2.94	4.65 ± 2.94
	Cervica	94.52 ± 3.69	5.48 ± 3.69
	Middle	96.28 ± 2.72	3.72 ± 2.72
	Apical	96.53 ± 3.72	3.47 ± 3.72
CEC (single-rooted premolars)	All	96.78 ± 1.81	3.23 ± 1.81
	Cervical	96.83 ± 1.76	3.17 ± 1.76
	Middle	97.36 ± 2.73	2.64 ± 2.74
	Apical	98.29 ± 1.51	1.71 ± 1.51
TEC (two dental roots premolars)	All	91.59 ± 3.48	8.41 ± 3.48
	Cervical	89.82 ± 4.1	10.18 ± 4.1
	Middle	95.34 ± 3.27	4.66 ± 3.27
	Apical	95.07 ± 5.13	4.93 ± 5.13
CEC (two dental roots premolars)	All	91.07 ± 6.38	8.93 ± 6.38
	Cervical	88.45 ± 9.06	11.55 ± 9.06
	Middle	93.75 ± 5.55	6.25 ± 5.55
	Apical	96.87 ± 3.49	3.13 ± 3.49

*CEC* contracted endodontic cavity, *TEC* traditional endodontic cavity

No statistically significant difference was observed between groups throughout the canal in each region (*p* > .05)

**Table 3:** Load at fracture (mean values ± standard deviations) for premolars with CEC or TEC assessed in the Instron Universal Testing Machine

Tooth type (*n* = 10)	UCW (% of total canal wall surface)		***P*** value
	CEC	TEC	
Single-rooted premolars	926.90 ± 194.97	888.57 ± 165.73	0.64
Two dental root premolars	665.09 ± 168.74	630.95 ± 159.81	0.88

## Discussion

Mininally invasive endodontics is widely accepted nowadays and attracted extensive attention. With the development of advanced equipments and techniques, such as CBCT, dental microscope, nickel-titanium instrument, as well as the single-cone technique. According to the concept, tooth structure preservation turn out to be the prime concern during an endodontic procedure. Recently, contracted endodontic cavity has been put forward to protect dental tissue as much as possible. However, this may lead to difficulty in operating due to the small operating space, insufficient visible light, and may also cause apical transportation, ledge, and instrument fracture. By analysis and compare systematically for the impact of contracted endodontic cavities and traditional endodontic cavities in premolars, the present study aims to provide references and advice to clinicians.

The result suggested that apical transportation after instrumentation for CEC was significantly bigger (*P* < 0.05) than TECs in premolars with two dental roots, and no instrument fracture was experienced, which means although the apical transportation of CEC was bigger in premolars with two dental roots, with the appropriate straight path to a root canal, there is a small probability that instrument fracture occurred in premolars. There was no significant difference (*P* > 0.05) between the TEC and CEC groups in the increased sectional area and the deviation of the central point in single-rooted premolars, which means it is safe and effective in single-rooted teeth.

Contracted endodontic cavities (CECs), as an alternative to traditional endodontic cavities, have been researched widely. So far, the outcomes and fracture resistance of CECs on root canal preparation still limited and controversial. Some studies showed that there was no difference between CECs and TECs in mean failure load in maxillary molars [22]. The type of access cavity does not influence the amount of remaining pulp tissue in root canals and isthmus in maxillary first molars [23]. Similarly, TECs lead to better preservation of the original canal anatomy in maxillary first permanent molars [24]. Most of the previous researches were focused on the efficacy of CECs and TECs treatment in maxillary molars, rarely in premolars. Firstly, the anatomy of premolars is complex and variation; secondly, the concentration of masticatory forces in premolars is big and the cervical of premolars is small; last but not least premolars are more likely to break after root canal therapy; therefore, minimally invasive endodontics in premolars is of great significance.

It is reported that guided endodontics printed templates have been used to locate all root canals in the apical third of teeth with pulp canal calcification and apical pathology with the aid of 3D printing technology and digital dentistry [16, 21]. In this research, we used guided endodontics printed templates for minimal cavity access, which acquired the least tooth structure removal and projected the access trajectory to each canal orifice. Contracted endodontic cavity with the aid of 3D-printed template seems to be a safe, clinically feasible method for locating root canals.

A previous study reported that CEC seems to exhibit better preservation of the original canal anatomy, particularly at the crown level, including incisors, premolars, and molars with TEC [25]. The conservative endodontic cavity, which could keep back the truss of dentin between the cavities, could save more dental tissue in premolars with two dental roots. Although more tooth tissue was retained, there was no obvious increase in the fracture resistance. It is probably because the volume of premolars themselveswas small, the increased fracture resistance of reserved coronal dentin could not offset the decreased amount that caused by RCT. Hence, further studies and a larger amount of simples are needed.

According to Neelakantan’report, Root canal was divided into coronal, middle and apical apart [18]. In the present research, due to similar sample size, it could avoid the difference of measurement and analysis caused by the differences of sample length.

Canal and crown boundaries were demarcated at the buccal-lingual level of the cementoenamel junction in single-rooted premolars, and canal boundaries were demarcated in root separation in premolars with two dental roots, all analyses were calculated separately for the cervical, middle, and apical thirds of the canal. Micro-CT allowed the 3D anatomy assessment of root canal fillings and voids, the results obtained in our study did not show obvious differences in the percentage of root canal filling in the CEC and TEC groups. Although the entrance of the pulp chamber is smaller in CECs, with the straight-line pathways into canals, the root canal filling can be completed for both CECs and TECs equally.

Numerous studies provided CEC preparation did not increase the fracture strength of teeth compared with TEC preparation [26–28]. This result corroborate with those above research. The researchers have found the endodontic procedures do not weaken teeth with intact marginal ridges [29], the CEC and TEC groups were both prepared with intact marginal ridges, and there were no significant differences (*P* > 0.05) on biomechanical responses between the premolars in the CEC and TEC groups. At the same time, the result was opposite to Krishan’s research [30], which reported CEC increased fracture resistance in premolars and mandibular molars. The following 4 explanations clarify this contradiction: (1) the simulated clinical treatment procedure to restore the access cavities with resin before fracture resistance test; (2) the angle of the tooth loaded at the central fossa from the tooth long axis and the spherical crosshead was different in this study; (3) single-rooted premolars and premolars with two dental roots were differentiated; (4) each sample has been tested by fatigue cycle test. All of these factors have a potential effect on the final results. In addition, the load type was comparable to that experienced in the mouth, and human teeth were subjected to forces in different directions at the same time, the tested teeth have irregular shapes, and the experimental data acquired were just in one direction, and the results were for reference. Besides, when we discuss about CECs, the extended preparation time and materials should be considered.

## Conclusions

Within the limitations of this study, the current results did not show obvious benefits associated with the CEC group compared with the TEC group. Although CECs could conserve more tooth hard tissue, the results of this study did not suggest that CEC could improve the fracture resistance of the endodontically treated premolars. The instrumentation efficacy and the percentage of filling material did not significantly differ between CECs and TECs in the premolars. Future experiments with bigger sample sizes and long-term clinical studies are encouraged to carry out on this topic.

Besides, contracted endodontic cavity with the aid of 3D-printed template seems to be a safe, clinically feasible method for locating root canals, which could be a prosperous future in minimally invasive endodontics.

## Data Availability

The datasets used and/or analysed during the current study are available from the corresponding author on reasonable request.

## Abbreviations

CEC: Contracted endodontic cavity
TEC: Traditional endodontic cavity
3D: Three-dimensional
MIE: Minimally invasive endodontics
PCD: Pericervical dentin
CBCT: Cone-beam computed tomography
RCT: Root canal therpy
Micro-CT: Micro computed tomography
